# Ginseng Berry Extract Prevents Atherogenesis via Anti-Inflammatory Action by Upregulating Phase II Gene Expression

**DOI:** 10.1155/2012/490301

**Published:** 2012-11-25

**Authors:** Chun-Ki Kim, Dong Hui Cho, Kyu-Sun Lee, Dong-Keon Lee, Chan-Woong Park, Wan Gi Kim, Sang Jun Lee, Kwon-Soo Ha, Oh Goo Taeg, Young-Guen Kwon, Young-Myeong Kim

**Affiliations:** ^1^Vascular Homeostasis Laboratory and Department of Molecular and Cellular Biochemistry, School of Medicine, Kangwon National University, Gangwon-do, Chuncheon 200-701, Republic of Korea; ^2^Department of Surgery, Seoul Medical Center, Seoul 131-130, Republic of Korea; ^3^Amorepacific Corporation R&D Center, Gyeonggi-do, Yongin 446-729, Republic of Korea; ^4^Division of Molecular Life Sciences, Ewha Womans University, Seoul 120-750, Republic of Korea; ^5^Department of Biochemistry, College of Sciences, Yonsei University, Seoul 120-749, Republic of Korea

## Abstract

Ginseng berry possesses higher ginsenoside content than its root, which has been traditionally used in herbal medicine for many human diseases, including atherosclerosis. We here examined the antiatherogenic effects of the Korean ginseng berry extract (KGBE) and investigated its underlying mechanism of action *in vitro* and *in vivo*. Administration of KGBE decreased atherosclerotic lesions, which was inversely correlated with the expression levels of phase II genes to include heme oxygenase-1 (HO-1) and glutamine-cysteine ligase (GCL). Furthermore, KGBE administration suppressed NF-**κ**B-mediated expression of atherogenic inflammatory genes (TNF-**α**, IL-1**β**, iNOS, COX-2, ICAM-1, and VCAM-1), without altering serum cholesterol levels, in ApoE^−/−^ mice fed a high fat-diet. Treatment with KGBE increased phase II gene expression and suppressed lipopolysaccharide-induced reactive oxygen species production, NF-**κ**B activation, and inflammatory gene expression in primary macrophages. Importantly, these cellular events were blocked by selective inhibitors of HO-1 and GCL. In addition, these inhibitors reversed the suppressive effect of KGBE on TNF-**α**-mediated induction of ICAM-1 and VCAM-1, resulting in decreased interaction between endothelial cells and monocytes. These results suggest that KGBE ameliorates atherosclerosis by inhibiting NF-**κ**B-mediated expression of atherogenic genes via upregulation of phase II enzymes and thus has therapeutic or preventive potential for atherosclerosis.

## 1. Introduction 

Although hyperlipidemia plays an important role in the pathogenesis of atherosclerosis [1], it is currently regarded as an inflammatory response of endothelial cell dysfunction at the initiation of atherosclerotic plaque formation [2]. Numerous evidences demonstrate that the pharmacological effects of statins, well-known antiatherogenic drugs on cardiovascular disease are closely correlated to their lipid-lowering and anti-inflammatory activities [3]. Recent studies demonstrated that anti-inflammatory compounds prevent the development of atherosclerosis, without altering blood lipid profiles in hyperlipidemic mice [4, 5], indicating that anti-inflammatory compounds may be used as therapeutic drugs for the treatment of atherosclerosis. 

Inflammatory genes, such as tumor necrosis factor-*α* (TNF-*α*), interleukin-1*β* (IL-1*β*), inducible nitric oxide synthase (iNOS), and cyclooxygenase-2 (COX-2), are highly expressed during atherosclerotic development [2, 6]. Of these gene products, iNOS and COX-2 synthesize nitric oxide (NO) and prostaglandin E_2_ (PGE_2_), respectively, and are involved in the pathogenesis of atherosclerosis. However, TNF-*α* and IL-1*β* promote the expression of intercellular adhesion molecule-1 (ICAM-1) and vascular cell adhesion molecule-1 (VCAM-1) in endothelial cells in atherosclerotic lesions [7]. Moreover, the interaction between endothelial cells and monocytes resulted in an increase in the transmigration of circulating monocytes to the intimal area of the vascular walls. Importantly, the expression of these genes is tightly regulated by activation of the transcription factor nuclear factor-*κ*B (NF-*κ*B) [8]. 

NF-*κ*B is a key player in inflammation gene expression and has a crucial role in atherosclerosis. In resting cells, NF-*κ*B exists in a heterodimeric inactive form in the cytosol bound to the distinct inhibitor *κ*B (I*κ*B). Inflammatory stimulants activate the I*κ*B kinase (IKK), which induces phosphorylation and proteasomal degradation of I*κ*B. Liberated NF-*κ*B translocates from the cytosol to the nucleus, where it promotes transcriptional induction of various inflammatory genes [8]. Recent studies have also demonstrated that herbal medicines and natural compounds can regulate NF-*κ*B-mediated inflammation-associated gene expression by upregulation of phase II antioxidant enzymes, including heme oxygenase-1 (HO-1) and glutamine-cysteine ligase (GCL) [9, 10]. Importantly, these enzymes inhibit intracellular accumulation of reactive oxygen species (ROS) involved in redox-dependent NF-*κ*B activation [11]. 

The root of *Panax ginseng* contains ginsenosides as active components, which are major contributors to its pharmacological activities, including anti-inflammatory and antiatherosclerotic effects [12]. These components are distributed in many parts of the ginseng plant, including the root, berry, and leaf. Different parts of the plant contain distinct ginsenoside profiles [13], and these parts may possess different pharmacological activities. Recent studies demonstrate that ginseng berry exhibits more potent antihyperglycemic activity and antiobesity effects in a mouse model than those of its root [14, 15]. Indeed, the ginseng berry has a different ginsenoside profile and higher ginsenoside content than its root [16]. Thus, the ginseng berry may exert more potent pharmacological effects on various human diseases than those of its root. However, the pharmacological effects of ginseng berry on atherosclerosis have not been studied.

The current study sought to determine if the Korean ginseng berry extract (KGBE) regulates atherosclerosis and to identify its underlying mechanism. We herein, demonstrate that KGBE suppressed atherosclerotic lesion development by inhibiting NF-*κ*B-mediated atherogenic inflammatory gene expression via the induction of the phase II antioxidant enzymes HO-1 and GCL, without lowering serum lipid levels, in a hyperlipidemic mouse model. These results provide new insights into the therapeutic potential of KGBE for the treatment of atherosclerosis.

## 2. Materials and Methods

### 2.1. Reagents

Dulbecco's modified Eagle's medium (DMEM), penicillin, and streptomycin were purchased from Life Technology Inc., (Rockville, MD, USA). Antibodies for p-I*κ*B*α* (sc-7977), I*κ*B*α*, NF-*κ*B p65, iNOS, ICAM-1, VCAM-1, TNF-*α*, COX-2, and IL-1*β* were obtained from Santa Cruz Biotechnology (Santa Cruz, CA, USA). Antibodies for HO-1, p-IKK*α*, and p-IKK*αβ* were obtained from Cell Signaling Technology (Beverly, MA, USA). TNF-*α*, vascular endothelial growth factor (VEGF) and ELISA kits for TNF-*α*, IL-1*β* and PGE_2_ were purchased from R&D Systems (Minneapolis, MN, USA). Calcein-AM and 2′,7′-dichlorofluorescin diacetate (DCFH_2_-DA) were purchased from Molecular Probes (Eugene, OR, USA). KGBE was prepared as indicated in the following procedure. Other chemicals were purchased from Sigma (St. Louis, MO, USA) unless indicated otherwise. 

### 2.2. Preparation of Korean Ginseng Berry Extract (KGBE) and Korean Red Ginseng Extract (KRGE)

**Figure 1:**
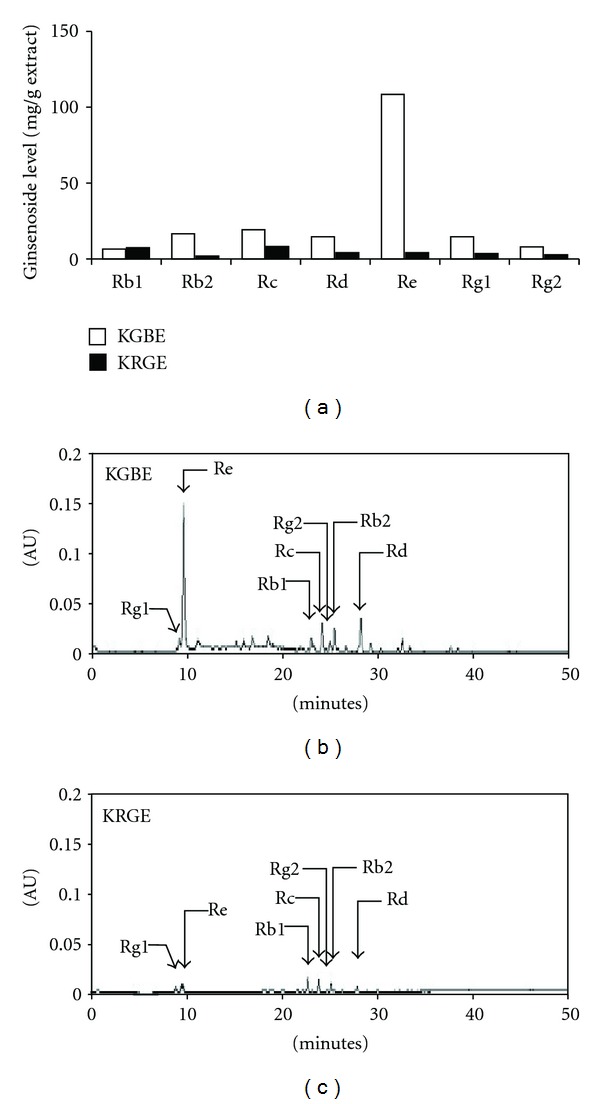
Major ginsenoside components and HPLC chromatograms of KGBE and KRGE. (a) Seven major ginsenosides were analyzed in KGBE and KRGE through HPLC analysis. (b, c) Representative HPLC chromatograms were obtained in KGBE and KRGE, respectively.

Korean ginseng berry was harvested, and the seeds were separated and removed. Next, the remnants were dried in hot air and refluxed with 70% ethanol for 10 h. After the extract was filtered and concentrated under reduced pressure at 45°C, KGBE powder was finally obtained by lyophilization and stored at −20°C until use. On the other hand, Korean red ginseng was extracted with reflux with 70% ethanol for 10 h. The extract was filtered according to a conventional method and concentrated under reduced pressure. KRGE powder was obtained by lyophilization and stored at −20°C until use. The lyophilized extracts were dissolved in culture medium before use and mixed with mouse chow diets. For comparison and standardization of ginsenoside composition, seven major ginsenosides were analyzed in KGBE and KRGE through HPLC. Ginsenoside compositions and HPLC chromatograms of both extracts were summarized in Figures 1(a)–1(c).

### 2.3. Cell Culture

Peritoneal macrophages were collected from the peritoneal cavity of 7-week-old male ApoE^−/−^ mice (Japan-SLC, Shizuoka, Japan) given an IP injection of 1.5 mL of 4% thioglycollate broth 7 days before harvest. Peritoneal macrophages and RAW264.7 cells were cultured in DMEM (2 mM L-glutamine, 100 units/mL penicillin, and 100 mg/mL streptomycin) containing 5% fetal bovine serum (Hyclone Labs, Logan, UT, USA) at 37°C in 5% CO_2_/95% air. Cells were pretreated with KGBE in the presence or absence of 20 *μ*M tin protoporphyrin IX (SnPP) and 20 *μ*M buthionine sulfoximine (BSO) for 30 min, followed by stimulation with LPS (100 ng/mL). Human umbilical vein endothelial cells (HUVECs) isolated from human umbilical cord veins were cultured as previously described [18] and used for experiments in passages 3 to 6.

### 2.4. Western Blot Analysis

Whole-cell lysates as well as cytosolic and nuclear fractions were prepared as previously described [17]. Samples (30 *μ*g protein) were separated on SDS-PAGE and transferred onto nitrocellulose membranes. The membranes were blocked with 5% nonfat-dried milk and hybridized with primary and secondary antibodies against target proteins. Protein bands were visualized by incubating membranes with chemiluminescent solution for 2 min and exposure to X-ray film.

### 2.5. Reverse Transcription-Polymerase Chain Reaction (RT-PCR)

RT-PCR was performed as described in a previous method [17]. Amplification conditions were as follows: denaturation at 94°C for 5 min for the first cycle and for 45 s starting from the second cycle, annealing at 56°C for 30 s for HO-1, GCL-catalytic subunit (GCLC), and *β*-actin. Final extension was performed at 72°C for 10 min. The primers used were 5′-GGCCCTGGAAGAGGAGATA-3′ (sense) and 5′-GCTGGATGTGCTTTTGGTG-3′ (antisense) for the HO-1, 5′-CACTGCCAGAACACAGACCC-3′ (sense) and 5′-ATGGTCTGGCTGAGAAGCCT-3′ for the GCLC and 5′-TCCTTCGTTGCCGGTCCACA-3′ (sense) and 5′-CGTCTCCGGAGTCCATCACA-3′ (antisense) for the *β*-actin. The amplification conditions and primer sequences for TNF-*α*, IL-1*β*, iNOS, COX-2, ICAM-1, and VCAM-1 were the same as previously described [17, 18]. The PCR products were separated on a 1% agarose gel and stained with ethidium bromide.

### 2.6. Analysis of NF-*κ*B Activation Pathway

Cells were pretreated with 100 *μ*g/mL KGBE and inhibitors (20 *μ*M SnPP and 20 *μ*M BSO) for 30 min and then stimulated with 100 ng/mL LPS for 30 min in the presence or absence of the proteasome enzyme inhibitors and 5 *μ*M MG132. Phosphorylation of IKK and I*κ*B and nuclear translocation of the NF-*κ*B p65 subunit were examined by Western blotting and immunohistochemical methods as described previously [17]. Electrophoretic gel mobility shift assay (EMSA) was performed for the measurement of NF-*κ*B-DNA-binding activity using ^32^P-labeled double-stranded NF-*κ*B-specific oligonucleotide and nuclear extracts [17]. Supershift and competitive assays were performed following preincubation of the nuclear extracts with 2 *μ*g of NF-*κ*B p65 antibody or an excess (100-fold) of cold probe at room temperature for 30 min. For assay for promoter activity, cells were transfected with piNOS-Luc or pNF-*κ*B-Luc in combination with pcDNA3-*β*-galactosidase using a lipofectamine method as described previously [17]. The cells were cultured for 20 h in fresh medium containing 5% fetal bovine serum and exposed to KGBE for 4 h, followed by stimulation with LPS for 12 h. Luciferase activity was measured in cell lysates by luminometer. Relative luciferase activity was calculated by normalization with *β*-galactosidase.

### 2.7. Monocyte-Endothelial Cell Interaction Assay

 Monocytic U937 cells were labeled with 5 *μ*M Calcein-AM in RPMI 1640 containing 10% FBS at 37°C for 1 h and washed twice with PBS by centrifugation. HUVECs were stimulated with 10 ng/mL TNF-*α* in 24-well plates for 8 h and then incubated with labeled monocytes (1 × 10^6^ cells/mL) at 37°C for 30 min. Nonadherent cells were removed by washing with RPMI 1640, and the plates were photographed by fluorescence microscopy. Monocytes bound to HUVECs were lysed with 50 mM Tris-HCl (pH 8.0) buffer containing 0.1% SDS. Fluorescent intensity was measured at excitation/emission wavelength of 485/535 nm using a florescence plate reader.

### 2.8. Animal Experiments

ApoE^−/−^ mice (C57BL/6J background, 6 to 7 weeks old, Japan-SLC, Shizuoka, Japan) were obtained from Orient (Sungnam, Korea) and maintained at the specific pathogen-free housing facility. All animal study protocols were approved by the Institutional Animal Care and Usage Committee of the Ewha Womans University (Seoul, Korea). Mice were divided randomly into four groups (*n* ≥ 10): (1) normal chow diet (NCD), (2) high-fat diet (HFD) alone, (3) HFD supplemented with 0.05% of lyophilized KGBE (W/W), and (4) HFD supplemented with 0.075% of lyophilized KRGE (W/W). HFD contains 0.15% cholesterol, 20% fat, and 0.05% sodium cholate. Mice were housed under 12 h light/12 h dark conditions. Bedding was changed once a week, and the temperature and humidity were controlled. The mice were given water and food *ad libitum*. Body weights and food intake were measured weekly at regular times. After 16 weeks, mice were sacrificed by cervical dislocation, and the serum levels of total cholesterol (TC), high-density lipoprotein-cholesterol (HDL-C), low-density lipoprotein-cholesterol (LDL-C), and triglyceride were measured using a 7020 Automatic Analyzer (Hitachi, Japan).

### 2.9. Analysis of Atherosclerosis in Atherosclerotic Mice

After mice were euthanized with carbon dioxide inhalation, the right atria were removed, and hearts and aortas were perfused with phosphate-buffered saline through the left ventricle. Analyses of *en face* and aortic sinus plaque lesions were performed as described previously [19]. 

### 2.10. Measurements of Nitric Oxide Metabolites, ROS, Cytokines, and PGE_2_

Nitrite and nitrite plus nitrate (NO_*x*_), stable oxidized products of NO, were measured in the culture media and serum using Griess reagents and a reductase-based colorimetric assay kit (Alexis San Diego, CA), respectively [17]. TNF-*α*, IL-1*β* and PGE_2_ levels were determined in sera and culture media using ELISA kits. Macrophages were pretreated with 100 *μ*g/mL KGBE in the presence or absence of 20 *μ*M SnPP and 20 *μ*M BSO for 30 min, followed by stimulation with LPS for 1 h. Intracellular ROS accumulation was determined using the H_2_O_2_-sensitive fluorescent dye, DCFH_2_-DA [17].

### 2.11. Statistical Analysis

All data are presented as the mean±standard deviation (SD) of at least three independent experiments. Student's *t*-test was also performed to identify which group difference accounted for the significant overall analysis of variance. *P* < 0.05 was considered statistically significant.

## 3. Results

### 3.1. KGBE Prevents Atherogenesis without Improving Serum Lipid Profiles in ApoE^−/−^ Mice

**Figure 2:**
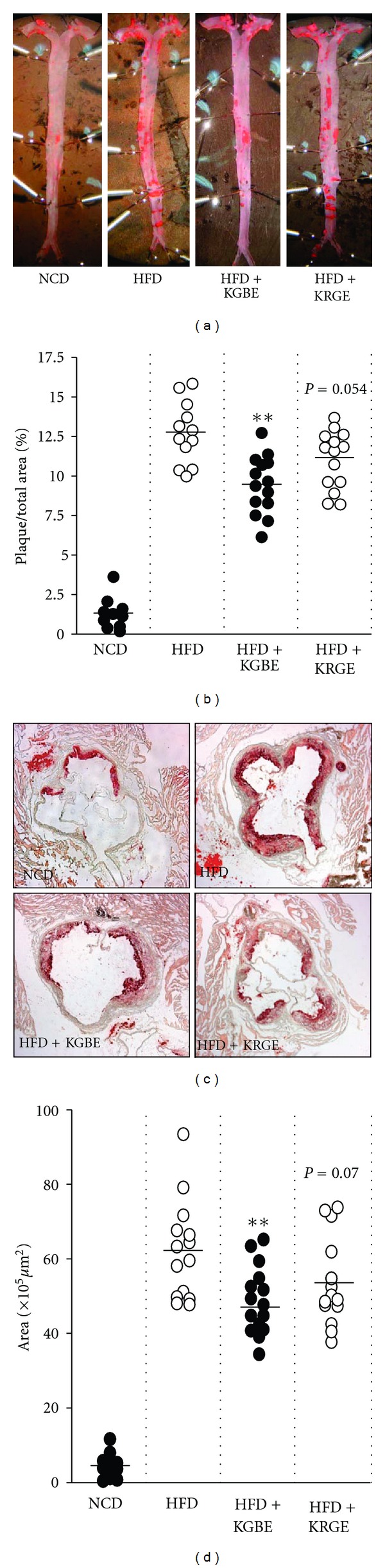
KGBE reduces atherosclerotic lesion formation in ApoE^−/−^ mice fed a HFD for 16 weeks. Mice were freely given NCD, HFD, and HFD supplemented with KGBE or KRGE for 16 weeks. (a) Pictures are representative *en face* images showing oil red O-stained plaque areas of aortas, and (b) relative plaque areas were quantified by computer-associated morphometry. Data shown are the mean ± S.D. (*n* ≥ 10). **P < 0.01 versus HFD. (c) Frozen sections of aortic sinus were stained with oil red O, and (d) plaque areas were quantified by computer-associated morphometry. Data shown are the mean ± S.D. (*n* ≥ 10). **P < 0.01 versus HFD.

We analyzed major seven saponin components in KGBE and KRGE extracts. Of them, the six ginsenosides, Rb2, Rc, Rd, Re, Rg1, and Rg2, showed high levels in KGBE as compared with those of KRGE, which is a well-known ginseng product (Figure 1(a)). The extraction yields of KGBE and KRGE were 22.8% and 47.9%, respectively. We first investigated whether KGBE exerts an antiatherosclerotic effect in HFD-fed ApoE^−/−^ mice by quantifying the atherosclerotic lesion area. Mice fed a HFD for 16 weeks exhibited significantly increased atherogenic areas in the aortic arch and descending aorta as compared with mice fed a NCD. This increase was significantly decreased by supplementation with KGBE (*P* < 0.01) but not with KRGE (*P* > 0.05) (Figures 2(a) and 2(b)). In addition, HFD-fed mice developed significant plaque lesions in the intima of their aortic sinuses, compared with NCD-fed control mice, and this plaque lesions were statistically reduced by supplementation with KGBE (*P* < 0.01), but not with KRGE (*P* = 0.07) (Figures 2(c) and 2(d)). Taken together, these results indicate that KGBE elicits a higher antiatherogenic effect than KRGE. We also assessed whether KGBE regulates serum lipid levels in ApoE^−/−^ mice fed a HFD (Table 1). No significant changes were observed in triglyceride, TC, LDL-C, and HDL-C levels between mice fed HFD alone and HFD supplemented with KGBE or KRGE. These results suggest that KGBE prevents atherosclerotic development without lowering serum lipid levels. Although not shown, the body weight between mice fed a HFD alone and in combination with KGBE was not statistically different. In addition, no notable differences in liver damage were observed between the two groups as determined by serum levels of glutamate-oxaloacetate transaminase and glutamate-pyruvate transaminase (data not shown). 

### 3.2. KGBE Inhibits the Production of Inflammatory Mediators in HFD-Fed ApoE^−/−^ Mice

**Figure 3:**
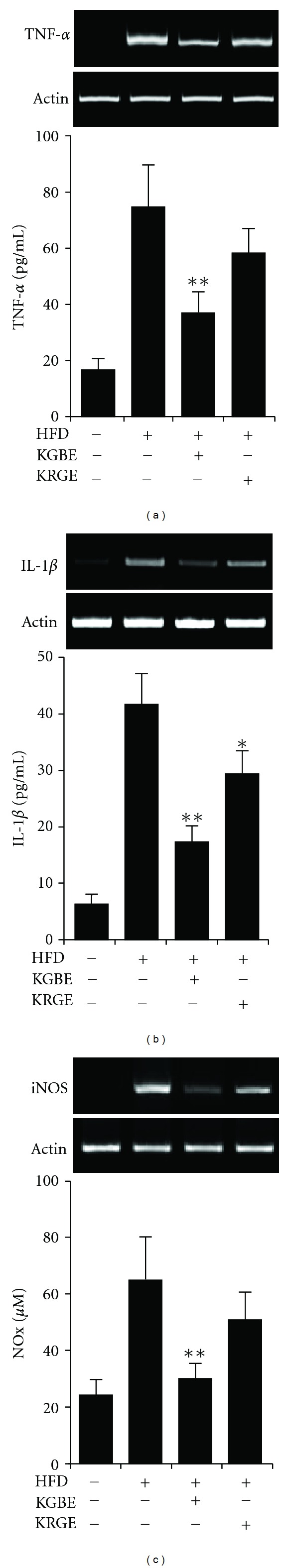
KGBE inhibits the production of inflammatory mediators in HFD-fed ApoE^−/−^ mice. (a–c, upper panels) Levels of TNF-*α*, IL-1*β*, and iNOS mRNAs were determined in the aortic tissues from mice fed NCD, HFD, and HFD supplemented with KGBE or KRGE for 16 weeks by RT-PCR. (a–c, lower panels) Levels of TNF-*α*, IL-1*β*, and NOx were determined in sera from mice using ELISA kits and a reductase-based Griess reaction kit. Data shown in graphs are the mean ± S.D. (*n* = 8). *P < 0.05 and **P < 0.01 versus HFD.

Monocytes/macrophages infiltrate atherosclerotic lesion areas and increase the expression of inflammatory genes, such as TNF-*α*, IL-1*β* and iNOS, which play an important role in development and progression of atherosclerosis [2, 6]. Thus, we next examined the effects of KGBE on the expression of these genes in aortic tissues and their gene product levels in sera. The levels of TNF-*α* and IL-1*β* mRNAs and their secreted proteins were significantly increased in aortic tissues and sera from mice fed a HFD, respectively, and these increases were reduced by supplementation with KGBE (Figures 3(a) and 3(b)). In addition, iNOS expression and NO production were also increased in aortic tissues and sera from mice fed a HFD, respectively, and these increases were significantly attenuated by KGBE (Figure 3(c)). Furthermore, all observed anti-inflammatory effects of KGBE were substantially higher than those of KRGE (Figures 3(a)–3(c)). 

### 3.3. KGBE Elicits the Anti-Inflammatory Effect in RAW264.7 Macrophage Cells

**Figure 4:**
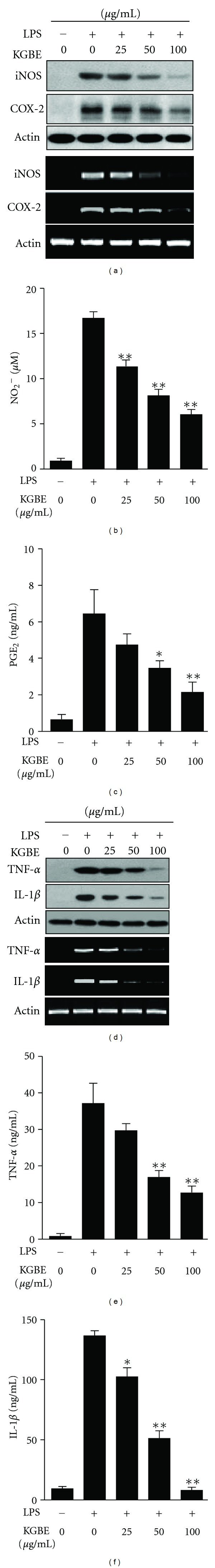
KGBE inhibits inflammatory gene expression in LPS-stimulated RAW264.7 cells. RAW264.7 cells were pretreated with the indicated concentrations of KGBE for 30 min. The cells were stimulated with 100 ng/mL LPS for 8 h (RT-PCR) and 12 h (Western blotting and medium analysis). (a, d) Intracellular protein and mRNA levels of inflammatory cytokines and iNOS were determined by Western blot and RT-PCR analyses. (b, c) Levels of nitrite and PGE_2_ were determined in the culture media using Griess reagents and an ELISA kit, respectively. (e, f) Levels of TNF-*α* and IL-1*β* were determined in the culture media using ELISA kits. Data shown in graphs are the mean ± S.D. (*n* = 4). *P < 0.05 and **P < 0.01 versus LPS.

Immune-activated macrophages express a variety of inflammatory genes, such as iNOS, COX-2, TNF-*α*, and IL-1*β*, which are major contributing factors in the pathogenesis of atherosclerosis [2, 6]. Experiments were performed to determine whether KGBE influences the expression of these genes in LPS-stimulated RAW264.7 cells. As expected, LPS-stimulated RAW264.7 cells increased expression of the inflammation-associated enzymes, iNOS and COX-2, as determined by Western blot and RT-PCR analyses, and these increases were inhibited in a dose-dependent manner by cotreatment with KGBE (Figure 4(a)). Similarly, treatment with KGBE inhibited LPS-induced production of nitrite (a stable oxidized product of NO) and PGE_2_ in a dose-dependent manner with an IC_50_ value of ~40 *μ*g/mL (Figures 4(b) and 4(c)). In addition, KGBE inhibited LPS-induced expression of the inflammatory cytokine genes, TNF-*α*, and IL-1*β*, in a dose-dependent manner (Figure 4(d)), and these inhibitory effects were highly correlated with increased levels of TNF-*α* and IL-1*β* in the culture media of LPS-stimulated RAW264.7 cells (Figures 4(e) and 4(f)). Under the same experimental conditions, KGBE did not reduce cell viability as measured by the crystal violet staining method (data not shown). These results suggest that KGBE regulates inflammatory gene expression in immune-activated macrophages.

### 3.4. KGBE Induces Phase II Enzymes in ApoE^−/−^ Mice and Primary Macrophages

**Figure 5:**
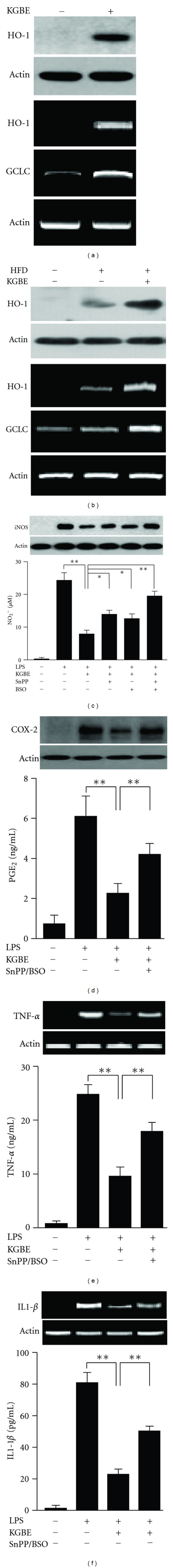
KGBE upregulates HO-1 and GCLC and inhibits inflammatory gene expression *in vitro* and *in vivo*. (a) Expression levels of HO-1 and GCLC proteins and mRNAs were analyzed in aortic tissues from ApoE^−/−^ mice fed NCD, HFD, and HFD supplemented with KGBE for 16 weeks. (b) Peritoneal macrophages isolated from ApoE^−/−^ mice were treated with 100 *μ*g/mL KGBE for 8 h, and levels of HO-1 and GCLC protein and mRNA were determined by Western blot and RT-PCR analyses. (c–f) ApoE^−/−^ macrophages were pretreated with 100 *μ*g/mL KGBE in the presence or absence of 20 *μ*M SnPP and 20 *μ*M BSO for 30 min. The cells were stimulated with LPS for 8 h (RT-PCR) and 12 h (Western blotting and medium analysis). Protein and mRNA levels of inflammatory cytokines and enzymes were determined by the methods described in Figure 3. Data shown in graphs are the mean ± S.D. (*n* = 4). *P < 0.05 and **P < 0.01.

Herbal medicines and natural compounds elicit anti-inflammatory activity through Nrf2-mediated upregulation of HO-1 and GCLC [9, 20]. As such, we next examined the *in vitro* and *in vivo* effect of KGBE on the expression of phase II genes and their role in KGBE-mediated anti-inflammatory activity. The expression of HO-1 and GCLC was slightly increased in aortic tissues from ApoE^−/−^ mice fed a HFD alone and further increased by supplementation with KGBE (Figure 5(a)). In addition, treatment with KGBE increased expression levels of HO-1 and GCLC in peritoneal macrophages isolated from ApoE^−/−^ mice (Figure 5(b)). Thus, we analyzed the functional role of these enzymes in KGBE-mediated anti-inflammatory activity using their respective inhibitors. The suppressive effects of KGBE on iNOS expression and NO production were reduced by co-treatment with either the HO-1 inhibitor SnPP or the GCL inhibitor BSO and synergistically reversed by combined treatment with both inhibitors (Figure 5(c)). These inhibitors also reduced the KGBE-mediated suppressive effects on COX-2 expression and PGE_2_ production in LPS-stimulated primary macrophages (Figure 5(d)). Moreover, treatment with SnPP and BSO together attenuated the inhibitory effects of KGBE on TNF-*α* and IL-1*β* mRNA expression and protein levels in the culture media of LPS-stimulated macrophages (Figures 5(e) and 5(f)). These results suggest that KGBE decreases inflammatory gene expression probably by upregulating HO-1 and GCLC.

### 3.5. KGBE Inhibits LPS-Induced ROS Generation and NF-*κ*B Activation by Increasing HO-1 and GCLC Expression

**Figure 6:**
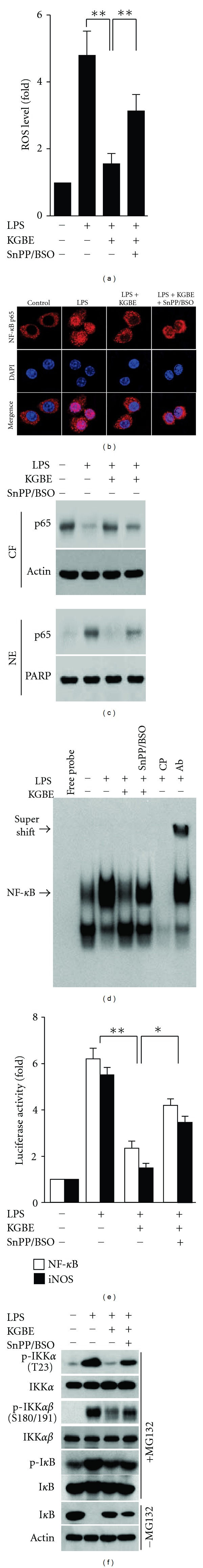
KGBE inhibits intracellular ROS levels and NF-*κ*B activation in LPS-stimulated ApoE^−/−^ macrophages. ApoE^−/−^ macrophages were pretreated with 100 *μ*g/mL KGBE in the presence or absence of 20 *μ*M SnPP and 20 *μ*M BSO for 30 min and stimulated with LPS for 1 h. (a) Intracellular ROS accumulation was determined using DCFH_2_-DA. (b, c) Nuclear translocation of NF-*κ*B was determined in whole cell mounts and nuclear and cytosolic fractions using confocal microscopy and Western blotting. (d) Nuclear NF-*κ*B-DNA-binding activity was determined in the presence or absence of cold probe (CP) or antibody for NF-*κ*B p65 (Ab) by EMSA. (e) Macrophages were transfected with piNOS-Luc or pNF-*κ*B-Luc using a lipofectamine method and stimulated with LPS for 16 h following pretreatment with KGBE alone or in combination with SnPP and BSO for 30 min. Luciferase activity was measured in cell lysates by a luminometer. (f) Macrophages were pretreated with KGBE alone or in combination with SnPP and BSO in the presence or absence of 5 *μ*M MG132 for 30 min, followed by stimulation with LPS for 30 min. I*κ*B phosphorylation and degradation and IKK phosphorylation were determined by Western blotting. Data shown in (a, e) are the mean ± S.D. (*n* = 3). *P < 0.05 and **P < 0.01.

Although high levels of ROS induce cytotoxicity, appropriate levels of ROS contribute to the activation of intracellular signal cascades, including NF-*κ*B pathway [21]. Therefore, we investigated whether KGBE regulates intracellular ROS levels in LPS-stimulated macrophages. KGBE effectively suppressed LPS-induced increases in intracellular ROS levels, and this effect was attenuated by combined treatment with SnPP and BSO (Figure 6(a)). We next examined the functional role of HO-1 and GCLC in KGBE-mediated regulation of NF-*κ*B activation. KGBE inhibited LPS-induced translocation of the cytosolic NF-*κ*B p65 subunit into the nucleus, thereby retaining its cytosolic level, and this inhibitory effect was reversed by combined treatment with SnPP and BSO, as determined by confocal microscopy and Western blot analysis (Figures 6(b) and 6(c)). We further confirmed the inhibitory effect of KGBE on the functional activation of NF-*κ*B by performing an EMSA. Treatment with KGBE suppressed LPS-induced increase in DNA-binding activity of NF-*κ*B in the nuclear extracts from macrophages stimulated with LPS, and this inhibitory effect was significantly reduced by combined treatment with SnPP and BSO (Figure 6(d)). The specific binding of NF-*κ*B to DNA was confirmed by a competitive assay with a cold probe and a supershift assay with an antibody against the NF-*κ*B subunit p65. We further examined the effect of KGBE on iNOS promoter activity and NF-*κ*B-Luc reporter activity. KGBE significantly inhibited LPS-induced increases in the transcriptional activities of iNOS promoter and NF-*κ*B-Luc construct, which were reduced by combined treatment with SnPP and BSO (Figure 6(e)). We next examined the effect of KGBE on the signaling cascade responsible for I*κ*B degradation. Treatment with KGBE suppressed LPS-induced phosphorylation of IKK*α* at threonine 23 and IKK*αβ* at serines 180/181, resulting in phosphorylation and degradation of I*κ*B, and these effects were attenuated by combined treatment with SnPP and BSO (Figure 6(f)). These results indicate that KGBE can inhibit LPS-induced NF-*κ*B activation by suppressing IKK-dependent I*κ*B degradation via upregulation of phase II enzymes and subsequent reduction of intracellular ROS levels. 

### 3.6. KGBE Inhibits the Expression of Adhesion Molecules in Endothelial Cells

**Figure 7:**
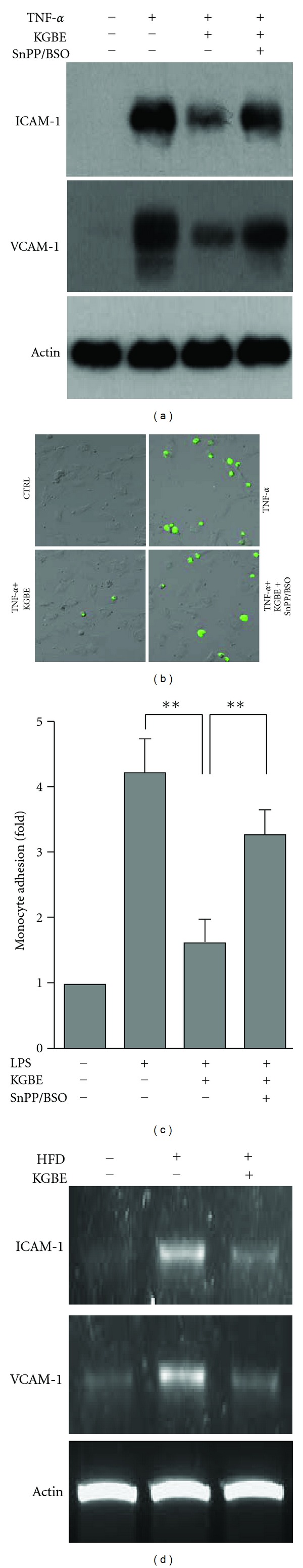
KGBE suppresses adhesion molecule expression in TNF-*α*-stimulated endothelial cells and aortas from mice fed a HFD. (a) HUVECs were pretreated with KGBE in the presence or absence of SnPP and BSO for 30 min. The cells were stimulated with 10 ng/mL TNF-*α* for 12 h. The protein levels of ICAM-1 and VCAM-1 were determined by Western blotting. (b) TNF-*α*-stimulated HUVECs were cocultured with fluorescence-labeled U937 cells for 1 h. After washing, adherent monocytes were photographed by fluorescence microscopy. (c) Monocytes bound to HUVECs were lysed, and fluorescence intensity was determined using a florescence plate reader. Data shown are the mean ± S.D. (*n* = 3). **P < 0.01. (d) Levels of ICAM-1 and VCAM-1 mRNAs were determined in aortic tissues from ApoE^−/−^ mice fed NCD, HFD, and HFD supplemented with KGBE for 16 weeks.

NF-*κ*B is also critical modulator of ICAM-1 and VCAM-1 expression, which are involved in vascular inflammation responsible for atherosclerotic development [22]. We hypothesized that KGBE could inhibit the expression of ICAM-1 and VCAM-1 via upregulation of HO-1 and GCLC. Thus, we examined the effect of KGBE on the expression of adhesion molecules in TNF-*α*-stimulated endothelial cells. As expected, KGBE treatment suppressed TNF-*α*-induced ICAM-1 and VCAM-1 expression in HUVECs, and its suppressive effect was significantly attenuated by treatment with both SnPP and BSO (Figure 7(a)). We next examined the effect of KGBE on leukocyte-endothelial cell interaction. KGBE effectively inhibited adhesion of leukocytes to HUVECs stimulated with TNF-*α*, and this inhibition was reversed by treatment with both SnPP and BSO (Figures 7(b) and 7(c)). We further investigated the *in vivo* effect of KGBE on ICAM-1 and VCAM-1 expression in aortic tissues of hyperlipidemic mice. ICAM-1 and VCAM-1 mRNA levels were significantly upregulated in aortic tissues from HFD-fed mice, and these increases were inhibited by administration of KGBE (Figure 7(d)).

## 4. Discussion

This study was undertaken to elucidate the pharmacological effect of KGBE on atherosclerotic development and investigate its underlying mechanism of action. Although administration of KGBE did not alter serum lipid contents, it resulted in a significant decrease in HFD-induced atherosclerotic lesion formation, which was highly correlated with its ability to upregulate the phase II antioxidant enzymes, HO-1, and GCLC. Furthermore, KGBE inhibited the expression of atherogenic inflammatory genes, such as TNF-*α*, IL-1*β*, iNOS, ICAM-1, and VCAM-1, in aortic tissues from mice fed a HFD. KGBE also elicited similar regulatory effects on the expression of phase II enzymes and atherogenic inflammatory genes in LPS-stimulated macrophages and TNF-*α*-activated endothelial cells. We also revealed that inhibition of both HO-1 and GCL activities effectively blocked KGBE-induced inhibitory effects on NF-*κ*B activation and atherogenic gene expression. These results suggest that KGBE prevents the pathogenesis of atherosclerosis by suppressing NF-*κ*B-mediated atherogenic inflammatory gene expression via upregulation of phase II antioxidant enzymes, without improving serum lipid profiles. 

The root of *Panax ginseng* is a well-known herbal medicine used to regulate vascular homeostasis and treat vascular diseases, including atherosclerosis and hypertension [23, 24]. Ginseng contains ginsenosides as active components, which contribute to its pharmacological activities [12]. The berry portion of ginseng also contains higher levels of ginsenosides than its root [16]. We also revealed that KGBE used in this study contains high levels of several ginsenosides, including Rb2, Rc, Rd, Re, Rg1, and Rg2. These evidences suggest that ginseng berry may exert more potent antiatherogenic effects on atherosclerosis than its root. We herein demonstrate that supplementation with KGBE (0.05%, W/W, *P* < 0.01), but not with KRGE (0.075%, W/W, *P* > 0.05), elicited the potent inhibitory effect on atherosclerotic plaque formation. It is well accepted that atherosclerosis develops as a consequence of high blood levels of cholesterol and triglyceride and chronic inflammatory responses. Although ginseng root extract is shown to lower total cholesterol and triglyceride levels in a hyperlipidemic rat model [25], our results show that KGBE has a greater suppressive effect on atherosclerotic lesion formation than Korean red ginseng extract (KRGE) in mice fed a HFD, without serum lipid-lowering activity. This different antiatherosclerotic effect between extracts of ginseng root and berry may be due to their distinct ginsenoside profiles [13]. These results suggest that the antiatherosclerotic effect of KGBE is not directly associated with its ability to lower blood cholesterol and lipid levels.

Atherosclerosis is a lipid-driven, chronic inflammatory disease of the vessel wall [2]. Immune cells and their mediators directly cause chronic arterial inflammation, which is a well-known factor in the pathogenesis of atherosclerosis. Indeed, anti-inflammatory compounds effectively inhibit atherosclerotic plaque formation in a hyperlipidemic animal model [4, 26]. Although our data did not indicate that KGBE reduces blood cholesterol levels, this extract effectively suppressed the serum levels of atherogenic inflammatory mediators, such as TNF-*α*, IL-1*β*, and NO, in mice fed a HFD. KGBE also attenuated the mRNA levels of these cytokines and protein levels of iNOS in aortic tissues from hyperlipidemic mice. These results suggest that KGBE suppresses atherosclerotic lesion formation by suppressing inflammatory gene expression, without lowering serum lipid levels. 

NF-*κ*B is a key regulator of inflammatory response, and its functional involvement in atherosclerosis is demonstrated by the presence of its activated form in human atherosclerotic plaques [5]. Furthermore, selective inhibition of NF-*κ*B reduces the area of atherosclerotic lesions [4]. Recent studies also demonstrated that anti-inflammatory compounds, which inhibit NF-*κ*B activation, prevent the development of atherosclerosis, without lowering serum lipid levels, in hyperlipidemic mice [4, 27]. In addition, the antiatherosclerotic effects of statins are associated with both the reduction of blood LDL-C levels and the suppression of NF-*κ*B-mediated inflammatory gene expression [3, 28]. These observations suggest that herbal and natural medicines that inhibit NF-*κ*B activation are beneficial in preventing atherosclerosis via suppression of atherogenic inflammatory gene expression. Atherosclerotic lesions are filled with various immune cells, such as monocytes and macrophages, which can orchestrate and elicit inflammatory responses, leading to the production of NF-*κ*B-mediated inflammatory genes [4]. We herein found that treatment with KGBE inhibited NF-*κ*B activation and suppressed the expression of the inflammatory cytokines and enzymes, TNF-*α*, IL-1*β*, iNOS, and COX-2 in LPS-stimulated macrophages. Of these gene products, TNF-*α* is an important contributor in the pathogenesis of atherosclerosis via leukocyte-endothelial cell interactions by upregulating the adhesion molecules ICAM-1 and VCAM-1 in endothelial cells [29], leading to the transmigration of circulating monocytes to the intima and formation of atheroma plaque [30]. We found that KGBE inhibited NF-*κ*B-mediated expression of ICAM-1 and VCAM-1 in TNF-*α*-stimulated HUVECs, resulting in the inhibition of monocyte-endothelial cell interactions. Moreover, administration of KGBE suppressed HFD-induced increases in adhesion molecule expression in a mouse model. These results suggest that KGBE inhibits the expression of atherogenic inflammatory genes via the suppression of NF-*κ*B activation.

**Figure 8:**
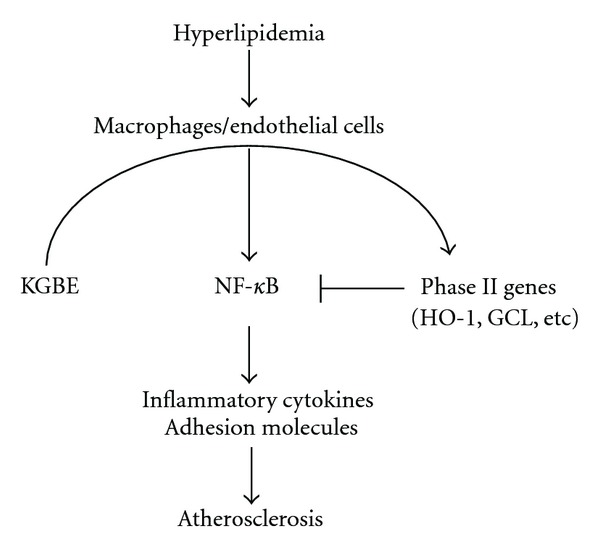
The possible antiatherogenic mechanism induced by KGBE.

It is well known that NF-*κ*B is activated by ROS and oxidized LDL, which play crucial roles in the initiation and progression of atherosclerosis [31]. ROS can indirectly elicit IKK-dependent NF-*κ*B activation by promoting redox-sensitive activation of the PI3K/PTEN/Akt and NIK/IKK pathways [17]. Our previous studies demonstrated that natural antioxidants effectively inhibit NF-*κ*B-mediated inflammatory gene expression by blocking intracellular ROS accumulation in LPS-stimulated macrophages [17, 32], implicating ROS as an important cellular signal mediator for NF-*κ*B activation. We here found that KGBE significantly reduced intracellular ROS levels, NF-*κ*B activation, iNOS promoter activity, and inflammatory gene expression in cultured macrophages stimulated with LPS. KGBE also inhibited phosphorylation of IKK*α* and IKK*αβ* and subsequent phosphorylation-dependent degradation of I*κ*B, which is an endogenous inhibitor of NF-*κ*B. These data indicate that KGBE inhibits atherogenic gene expression via suppression of the redox-sensitive NF-*κ*B activation pathway, particularly regulation of phosphorylation-dependent activation of IKK by decreasing intracellular ROS level (Figure 8).

An increasingly large body of evidence points to a crucial role of Nrf2-dependent phase II enzymes in anti-inflammation and anti-atherosclerosis [11, 20]. The anti-inflammatory effect of phase II antioxidant enzymes is associated with inhibition of NF-*κ*B activation by modulating ROS generation and removal. Of these enzymes, HO-1 and GCL are the most potent antioxidant enzymes. HO-1 catalyzes heme to biologically active products: carbon monoxide and biliverdin/bilirubin, which regulate inflammatory response by acting as potential antioxidants [33]. On the other hand, GCL, a rate-limiting enzyme in glutathione biosynthesis, elevates intracellular GSH levels and redox potential, which are likely to inhibit inflammation-associated gene expression by blocking the redox-sensitive NF-*κ*B activation pathway [34]. Furthermore, the natural compound carnosic acid elevates intracellular GSH levels via upregulation of Nrf2-dependent phase II enzyme GCL expression, leading to suppression of NF-*κ*B activation and inflammatory reaction [35, 36]. We here found that KGBE elevated the expression of HO-1 and GCL *in vitro* and *in vivo*. Inhibitors of HO-1 (SnPP) and GCL (BSO) significantly reversed the suppressive effects of KGBE on LPS-induced ROS generation, NF-*κ*B activation, and atherogenic inflammatory gene expression in cultured macrophages. In addition, these inhibitors effectively ameliorated the inhibitory effect of KGBE on adhesion molecule expression in TNF-*α*-stimulated endothelial cells. Of the ginsenosides detected in KGBE, Rb1, Rd, and Re are known to induce Nrf2-mediated phase II genes and/or inhibition of the NF-*κ*B pathway [37–40], suggesting these ginsenosides as possible candidates in the strong anti-atherogenic activity of KGBE. These results indicate that KGBE inhibits the expression of atherogenic inflammatory genes and adhesion molecules via upregulation of Nrf2-dependent antioxidant genes, HO-1 and GCL, which are responsible for the reduction of intracellular ROS levels (Figure 8). 

Although ginseng berry has not been shown to elicit anti-inflammatory and anti-atherogenic effects, we first revealed that KGBE elicited anti-atherogenic and anti-inflammatory effects, with upregulation of phase II antioxidant genes. Increasing evidences have been shown that Nrf2-dependent phase II gene expression plays an important role in preventing chronic inflammation and atherosclerotic pathogenesis [20, 41]. Therefore, activators of Nrf2 pathway that increases expression of phase II and antioxidant enzymes can be used as novel therapeutic strategies for restoring cellular redox homeostasis and diminishing NF-*κ*B-mediated inflammation in cardiovascular diseases, including atherosclerosis [42, 43]. Indeed, dietary and herbal Nrf2 activators result in the upregulation of multiple endogenous antioxidant mechanisms and decrease the possibility of developing atherosclerotic lesions [11, 41]. Our previous report also showed that siRNA-based Nrf2 activation suppresses vascular inflammation in a mouse model [44]. In addition, administration of an Nrf2 activator resulted in significant phase II gene upregulation and decreased atherogenesis in LDLR^−/−^ mice [45], ApoE^−/−^ mice [46], and rabbits fed a high-fat diet [47]. These evidences suggest that KGBE-induced increases in Nrf2-mediated phase II gene expression is linked to its medicinal properties with regard to anti-inflammation and anti-atherogenesis. Our results demonstrate that KGBE might prevent vascular inflammation and atherosclerotic development by inducing Nrf2-mediated phase II gene expression.

Our findings presented herein indicate that KGBE reduces HFD-induced atheromatous lesion formation in a mouse model via an anti-inflammatory mechanism by promoting the expression of HO-1 and GCL, which are responsible for inhibiting NF-*κ*B activation by reducing intracellular ROS levels. These findings suggest that KGBE inhibits the pathogenesis of hyperlipidemic atherosclerosis by suppressing NF-*κ*B-mediated expression of atherogenic inflammatory genes via induction of phase II antioxidant enzymes, such as HO-1 and GCL, without lowering serum lipid levels. Thus, KGBE could be considered as a potential therapeutic and preventive herbal medicine for the treatment of atherosclerosis and its related complications.

## Figures and Tables

**Table 1:** Effect of KGBE and KRGE supplementation on serum lipid levels in hyperlipidemic mice.

Lipids	NCD	HFD supplemented with		
		None	KGBE	KRGE
Total Cholesterol	448 ± 45	1105 ± 29	1072 ± 37	1097 ± 41
HDL-Cholesterol	34 ± 16	22 ± 10	25 ± 12	23 ± 13
LDL-Cholesterol	72 ± 42	261 ± 23	254 ± 48	247 ± 36
Triglyceride	86 ± 10	126 ± 23	119 ± 15	114 ± 16

Unit of all lipid contents in serum is mg/dL. NCD: normal chow diet; HFD: high-fat diet; KGBE: Korean ginseng berry extract powder (0.05%, W/W); KRGE: Korean red ginseng extract powder (0.075%, W/W).
